# Factors associated with systolic hypertension in peritoneal dialysis patients

**DOI:** 10.1007/s40620-019-00633-y

**Published:** 2019-08-10

**Authors:** Surachet Vongsanim, Andrew Davenport

**Affiliations:** 1 Renal Division, Department of Internal Medicine, Faculty of Medicine, Chiang Mai University, 110 Intrawarorot Road, Amphoe Mueang Chiang Mai, Chiang Mai, 50200 Thailand; 2 UCL Department of Nephrology, Royal Free Hospital, University College London, London, UK

**Keywords:** Systolic blood pressure, Extracellular water, Sodium, Peritoneal transport, Brain natriuretic peptide

## Abstract

**Background:**

Hypertension is common in peritoneal dialysis (PD) patients and associated with adverse outcomes. Besides solute clearance, PD convective clearance is used to control extracellular water (ECW) volume and sodium balance. Previous studies have reported on hypertension in PD patients treated with continuous ambulatory peritoneal dialysis (CAPD) using hypertonic glucose dialysates. However, increasing numbers of PD patients are now treated with automated peritoneal dialysis (APD) and icodextrin dialysates. As such, we wished to explore factors associated with systolic blood pressure (SBP) in a modern cohort to identify targets to improve blood pressure control in PD patients.

**Methods:**

We retrospectively reviewed the results from PD patients attending for peritoneal membrane assessment who had corresponding bioimpedance ECW and brain natriuretic peptide (NT-proBNP) measurements.

**Results:**

We studied 510 PD patients: 317 (72.2%) male, 216 (42.4%) diabetics, median age 59 (47–72) years, and 51% treated by APD with a day-time icodextrin exchange. Mean systolic blood pressure (SBP) was 140 ± 24.8 mmHg. SBP was independently associated with 4-hour dialysate to plasma creatinine ratio (β = 29.5 (95% confidence limits 11.4–47.5, p = 0.001), N-terminal brain natriuretic peptide [β = 11.9 (7.2–16.7), p < 0.001], and daily urine sodium excretion [β = 1.7 (1.0–2.3), p < 0.001].

**Conclusion:**

In the era of APD cyclers and icodextrin, SBP is associated with increased NT-proBNP, a marker of ECW expansion, and faster peritoneal transport, a risk factor for a positive sodium balance, and increased urinary sodium suggestive of higher dietary sodium intake. Patients should be encouraged to restrict sodium intake and PD prescriptions targeted to control ECW to improve SBP control.

## Introduction

Hypertension is one of the major determinants contributing to the increased cardiovascular morbidity and mortality in dialysis patients [1]. Moreover, hypertension is very common in patients with end-stage kidney disease treated with peritoneal dialysis (PD), affecting more than 80% of patients and observational studies reporting an association with worse long-term outcomes [2, 3]. The prospective Netherlands Cooperative Study on the Adequacy of Dialysis observed an association between high systolic blood pressure and an increased risk of mortality [4]. Current clinical guideline from the International Society of Peritoneal Dialysis (ISPD) recommends a target blood pressure of less than 140/90 mmHg as the treatment goal for PD patients [5]. However, this recommendation was based mainly on studies from the general and chronic kidney disease populations, rather than specifically from studies in PD patients.

Understanding the relationship between clinical factors affecting the blood pressure is the first step to improve treatment outcomes for individual patients. Although some studies have reported an association between volume overload, with extracellular water (ECW) expansion and hypertension [6, 7], whereas other reports failed to demonstrate any association between volume overload and hypertension [8], and others observed an effect of restricting dietary sodium intake [9]. The introduction of bioimpedance devices into clinical practice to measure ECW expansion and body composition [10], has led to the realization that ECW expansion can also occur in patients with malnutrition and inflammation [11], and this may aid explaining the discordant results of previous studies investigating the association between volume status and hypertension.

Many of these previous studies investigated patients treated by continuous ambulatory peritoneal dialysis (CAPD) prescribed hypertonic glucose peritoneal dialysates. In recent years, more patients in Europe and North America are treated using automated peritoneal dialysis cyclers (APD), rather than CAPD, and APD cyclers help reducing ECW expansion in faster peritoneal transporters [12, 13]. Similarly, the introduction of icodextrin peritoneal dialysates for the long dwell has been shown to reduce ECW expansion compared to hypertonic glucose [8].

We therefore wished to identify factors associated with systolic blood pressure in a modern day cohort of PD patients prescribed icodextrin dialysates for both CAPD and APD, that would aid understanding of pathophysiology of hypertension and generate hypotheses that could be tested and improve blood pressure control in PD patients.

## Study methods

We retrospectively reviewed the results from adult peritoneal dialysis patients who had attended for routine peritoneal membrane assessment in our centre between January 2008 and October 2018. No patient had been treated for PD peritonitis or had an emergency hospital admission within the preceding 3 months. Patients with a urinary output of ≥ 200 mL/day were prescribed frusemide 250 mg daily. We excluded patients who had implantable cardiac devices, amputations and those were unable to stand. Patients starting PD were provided with dietary advice to restrict dietary sodium to 100 mmol/day.

All patients used standard low pH glucose or 7.5% icodextrin dialysates (Baxter health Care, Deerfield, Illinois, USA). Patients were weighed and had bioimpedance measurements post voiding and with peritoneal dialysate drained out [14]. Peritoneal transport (PET) was calculated as the ratio of 4-hour peritoneal dialysate effluent creatinine to serum, using a 2 litres of 22.7 g/L glucose exchange [13]. PD adequacy and dietary protein nitrogen appearance adjusted for body weight (nPNA) were calculated by standard methods from 24-hour urine and peritoneal dialysate effluent samples [13]. In addition to standard blood tests, we also measured N-terminal brain natriuretic peptide (NT-proBNP). Sodium in urine and dialysates was measured using an indirect ion electrode [15]. CAPD patients were taught to allow 15 s for the flush before fill technique, and as such we allowed 90 mL to compensate for this when estimating sodium balance as the difference between the total amount of sodium instilled with PD dialysates and that measured in 24-hour PD effluent and urine collections. Multifrequency bioelectrical impedance assessments (MFBIA) were measured with an eight electrode multifrequency segmental bioimpedance device (InBody 720, Seoul, South Korea) using a standardised protocol, after the patient had drained out peritoneal dialysate. Extracellular water (ECW) and skeletal muscle mass (SMM) [16], were normalized by height and height squared, respectively, to allow comparison between patients. Patients were admitted for the peritoneal membrane assessment and blood pressure was recorded in the supine position after 4 hours when the patient had drained out their dialysate and then rested for a minimum additional 30 min and had abstained from any stimulants. Blood pressure measurements were repeated, and if similar the first blood pressure recorded, but if lower, then a third measurement was made, and the mean of the lower two recordings taken, following the British Society of Hypertension guidelines [17]. Blood pressure monitors (Dinamap, Critikon Corporation, Tampa, FL, USA) were regularly serviced and calibrated. Relevant medical history and echocardiography results were obtained from hospital computerised records. Left ventricular mass was calculated from 2-dimensional transthoracic echocardiograms (Philips IE33, Philips Medical Systems, Eindhoven, the Netherlands) and analysed offline by experienced observers using the equation of Devereux [18]. Left ventricular mass index (LVMI) was calculated as the left ventricular mass divided by body surface area (BSA) using the Gehan and George formula [19].

### Ethics

Our retrospective audit complied with the UK National Health Service guidelines for clinical audit and service development with all patient data anonymised and complied with UK National Institute for Clinical Excellence best practices, www.nice.org.uk/media/796/23/bestpracticeclinicalaudit.pdf.

### Statistical analysis

All categorical data are presented as percentage and continuous data as mean ± standard deviation, or median (interquartile range). Groups were compared by anova or Kruskal–wallis, for parametric and nonparametric data with appropriate post hoc testing. Univariate correlation was by Pearson or Spearman analysis for parametric and nonparametric data, respectively. Variables of interest (p < 0.1) were then entered into multivariable step-backward logistic regression model. Transformation of data was performed if required to improve variable distribution. Variables were then only retained where the 95% confidence intervals for the estimate did not include zero or there was an improvement in model fit (as demonstrated by the − 2log likelihood), models were checked for collinearity and variable inflation factor. Statistical analysis used Statistical Package for Social Science version 24.0 (IBM Corporation, Armonk, New York, USA). Statistical significance was taken as p < 0.05.

## Results

Five hundred and forty-nine patients started PD, 31 patients were unable to have bioimpedance measurements and 8 patients developed PD peritonitis shortly after starting PD or had an acute illness, and so we studied five hundred and ten adult PD patients attending the clinic for their first assessment of peritoneal membrane function 2 (2–3) months after starting PD, who had MFBIA recorded on the same day; (Table 1). Antihypertensive medications were prescribed to 80.8% of patients, median number of medications 1 (1–2), and diuretics to 84.6%.

**Table 1:** Patient demographic characteristics

Characteristics	Values
Male (%)	317 (72.2%)
Age, years	59 (47–72)
Diabetes mellitus (%)	216 (42.4%)
Icodextrin usage (%)	364 (71.4%)
PD mode (%)	
APD	138 (27.1%)
CAPD	113 (22.12%)
APD with day time exchange	259 (50.8%)
Office blood pressure, mmHg	
Systolic	140.9 ± 24.8
Diastolic	81.5 ± 15.3
Serum creatinine, ummol/L	566 (434–742)
Weekly Kt/Vurea	2.56 (2.06–3.29)
Urine volume, mL/day	1105 (573–1680)
Urinary sodium loss, mmol/day	59 (30–156)
Peritoneal sodium loss, mmol/day	47.7 (− 195 to 405)
PET 4-hour D/Pcreatinine	0.73 ± 0.13
Serum albumin, g/L	37 ± 4.5
nPNA, g/kg/day	0.87 (0.74–1.05)
NTproBNP, ng/L	2163.0 (837.2–7119.9)
Serum sodium mmol/L	139 (136–141)
Total body water, L	37.50 (31.30–43.43)
ECW/height, Lkg/m	8.98 ± 1.71
ECW/body weight L/kg	0.21 ± 0.09
SMM/height^2^, kg/m^2^	9.82 ± 1.60
LVEF, %	55.0 (45.0–57.5)
Left ventricular mass index, g/m^2^	112.5 (89.4–136.1)

Values presented as mean ± standard deviation, median (interquartile range) or number (percentage)

Data expressed as integer, percentage, mean ± standard deviation or median (interquartile range)

*APD* automated peritoneal dialysis cycler, *CAPD* continuous ambulatory peritoneal dialysis, *PET* peritoneal equilibration test, *D/P* 4 h dialysate to plasma ratio, *nPNA* normalised protein nitrogen appearance rate, *NTproBNP* N terminal brain natriuretic peptide, *ECW* extracellular water, *SMM* skeletal muscle mass, *LVEF* left ventricular ejection fraction

Three hundred and ninety-three (77.1%) had echocardiography reports.

We divided patients according to peritoneal transport status [13], patients who were faster transporters had higher systolic blood pressure (SBP), NTproBNP, and bothe EC and ECW adjusted for height compared to slower transporters (Table 2).

**Table 2:** Patients divided according to peritoneal creatinine transport status

Variables	Slow (15)	Slow average (119)	Fast average (235)	Fast (138)	p value
SBP mmHg	126 (115–152)	134(118–148)	144(125–158)	144(129–164)	0.001
DBP mm Hg	78 ± 13	81 ± 15	81 ± 15	82 ± 15	0.625
NTproBNP ng/L	1750 (829–3713)	1412 (465–4389)	2140 (899–6023)	3691 (1351–15,764)	< 0.001
Na Balance mmol/day	− 110.9 (133.6 to − 63.8)	− 114.1 (− 159.3 to − 78.3)	− 114.3 (− 178.1 to − 70.45)	− 136.9 (− 175.7 to − 82.7)	0.302
ECW L	12.1 (10.6–14)	13.3 (11.4–16.3)	15.2 (13.0–17.8)	15.35 (13.1–17.3)	< 0.001
ECW/Ht L/m	7.6 (6.5–8.9)	8.4 (7.2–9.6)	9.1 (8.0–10.4)	9.3 (8.1–10.1)	< 0.001
ECW/Wt L/kg	0.19 ± 0.04	0.20 ± 0.03	0.21 ± 0.04	0.22 ± 0.03	0.424

*SBP* systolic blood pressure, *DBP* diastolic blood pressure, *NTproBNP* N terminal brain natriuretic peptide, *ECW* extracellular water, *Ht* height, *Wt* weight

p value fast vs slow/slow average transporter

On univariate analysis SBP was positively associated with diabetes, serum creatinine, urine sodium, peritoneal membrane transporter status, NT-proBNP, ECW/height and SMM/height^2^ and negatively with serum albumin. We found no statistically significant association between left ventricular ejection fraction (LVEF), left ventricular mass index (LVMI) and SBP, prescription of, or the number of anti-hypertensive medications prescribed (Table 3).

**Table 3:** Univariate analysis of factors associated with systolic blood pressure

Variables	R	p value
Diabetes	0.105	0.018
Serum creatinine	0.125	0.005
Urine sodium (mmol/day)	0.171	< 0.001
4-hour D/Pcreatinine	0.209	< 0.001
Serum albumin (g/L)	− 0.169	< 0.001
NT-proBNP (pg/mL)	0.205	< 0.001
ECW/height (kg/m)	0.211	< 0.001
SMM/height^2^ (kg/m^2^)	0.195	< 0.001

N terminal brain natriuretic peptide (NT-proBNP), extracellular water (ECW), skeletal muscle mass (SMM)

A multivariable step backwards regression analysis including all factors that were significant on univariate analysis. SBP was independently associated with faster membrane transport, daily urine sodium excretion, and NT-proBNP (Table 4).

**Table 4:** Multivariable model for systolic blood pressure

Variables	β	StE β	St β	t	95% CI	p value
Diabetes mellitus	4.15	2.34	0.09	1.77	− 0.46 to 8.75	0.08
Urine sodium	1.65	0.34	0.246	4.86	0.98–2.32	< 0.001
4-hr D/Pcreatinine	29.46	9.17	0.159	3.21	11.44–47.48	0.001
NT-proBNP pmol/L	11.90	2.42	0.243	4.93	7.15–16.65	< 0.001
SMM/height^2^ kg/m^2^	1.35	0.77	0.089	1.758	− 0.16 to 2.87	0.08

Nonparametric data was transformed to obtain normal distribution by log transformation (NT-proBNP) or square root (Urinary sodium). Standard error β (StE β), Standardized β (Stβ), 95% confidence intervals (95% CI). Model fit r^2^ = 0.173, adjusted r^2^ 0.162

*NT*-*proBNP* N terminal brain natriuretic peptide, *SMM* skeletal muscle mass

## Discussion

The majority of the newly established PD patients in our study were fast average or fast transporters [13], with 50.8% patients treated with APD and a day time icodextrin exchange and 71.4% prescribed icodextrin as part of their PD prescription. As such, compared to previous reports, we report on an incident cohort of PD patients with the majority using icodextrin dialysates [2–4]. Icodextrin has been shown to improve volume control compared to hypertonic glucose and has been shown to have an important role in maintaining ultrafiltration, particularly for the faster transporter [20].

As expected, the majority of patients attending for their first assessment of peritoneal membrane function had residual renal function. Most of our patients had blood pressure measurements within the current ISPD guideline targets [5]. Compared to previous reports, our patients had lower LVMI and well preserved left ventricular function [21]. In view of the relatively short duration of treatment with PD, the lower levels of left ventricular hypertrophy reported in our study, this most probably reflects the standard of pre-dialysis care provided by a specialist clinical service, designed to prepare patients for dialysis. Although LVMI is associated with hypertension, we could not find any significant association with systolic blood pressure, diastolic blood pressure, pulse pressure, or mean arterial pressure. Other studies in PD patients have similarly found no association [22]. We used clinic blood pressures in our analysis, differing from some of the previous studies, but even those using ambulatory blood pressure recordings have reported no association between LVM and blood pressure [22]. Whereas studies in haemodialysis patients have reported on blood pressure variability, similar studies using ambulatory blood pressure monitoring have not shown such day to day variability [23].

On univariate analysis, we observed a negative association between SBP and serum albumin, whereas SBP was positively correlated with diabetes, serum creatinine, 24-hour urine sodium excretion, peritoneal membrane transport status, NT-proBNP, ECW/height and SMM/height^2^. After adjustment using a multivariable model, SBP was found to be independently associated with daily urine sodium excretion, 4-hour peritoneal membrane creatinine transport, and NT-proBNP.

Overhydration and hypertension are commonly reported in PD patients, and cardiovascular death is the commonest cause of mortality for PD patients [6, 24]. Volume overload has been shown to be an important factor associated with hypertension in PD patients, as better volume control has been observed to improve blood pressure control and reduce left ventricular hypertrophy [25, 26]. NT-proBNP is a cardiac biomarker secreted by cardiomyocytes in the ventricles in response to plasma volume expansion, and increased ECW [27, 28]. Studies in PD patients have reported an association between increasing NT-proBNP and ECW volume expansion and mortality [27, 28]. The association with SBP would support ECW being a contributor to an increased SBP.

Diabetes is a common comorbidity in patients with chronic kidney disease. Diabetic patients may have faster transport status [29], as hyperglycaemia can increase local blood flow by protein kinase C mediated vasodilatation. Faster peritoneal transporters are at risk of lower ultrafiltration volumes when longer dwell cycles are prescribed due to loss of the osmotic glucose gradient. Other studies have shown that diabetic patients have ECW expansion compared to non-diabetics, even after adjusting for transporter status [30]. Although the hyperglycaemic state may reduce the osmotic effects of the dialysate glucose and so reduce convective peritoneal sodium removal, diabetic patients have also been reported to have higher tissue sodium levels [31], and this may additionally contribute to an increased risk for hypertension.

Faster peritoneal membrane permeability has been reported associated with increased mortality, possibly due to ECW expansion [20]. Although faster transport status was associated with PD technique failure and increased mortality, this was in the era before APD cyclers and icodextrin [32]. Our study had a high proportion of patients prescribed icodextrin and APD cyclers, and yet we found that faster transport was still associated with increased SBP, ECW expansion and raised NTproBNP. Although APD cyclers allow faster transporters to achieve greater ultrafiltration, the shorter cycle dwell times result in a relative greater water transport through aquaporin channels compared to sodium removal by active Na/K ATPase transporters and Na co-transporters, so increasing the risk of sodium retention and systolic hypertension [33].

24-hour urine sodium has been used to estimate daily sodium intake particularly in patients with chronic kidney disease, on the basis that if patients are in neutral balance, then urinary sodium excretion should mirror dietary intake. However, this assumes that patients are in neutral balance and that 24-hour collections are both accurate and reliable. Dietary recall may also be inaccurate due to the increasing consumption of ready meals, and additions from pre-prepared sauces [34, 35]. As such we were unable to reliably estimate dietary sodium intake, and sodium balance in terms of dietary intake and urinary and peritoneal sodium removal. Previous studies have demonstrated that reduced sodium intake led to a significantly lower blood pressure in PD patients [9]. On the other hand one study reported that patients with faster transport had lower peritoneal sodium losses and higher SBP [32]. This is most likely due to our incident patients having greater dietary sodium intake, whereas in the study from Turkey of prevalent patients, using only glucose dialysates, failure to achieve adequate peritoneal sodium removal led to increased SBP and volume overload. As our patients with greater sodium losses had greater muscle mass, suggesting greater sodium intake in keeping with previous studies reporting greater survival for those patients with the highest sodium removal [36], and greater mortality for those with lowest dietary sodium intake [37].

Our study was a cross sectional observational study in an incident cohortand as such we cannot attribute causality as to which factors increase SBP, but provide hypotheses which require testing. However the association with increased NTproBNP and ECW is suggestive that increased blood pressure was volume related, particularly when considering that our faster peritoneal transporters have higher SBP, NT-proBNP and ECW.

Our study of PD patients in the modern era of APD cyclers and icodextrin supports earlier reports that SBP in PD patients is associated with faster peritoneal transport, which is a risk factor for increased ECW and sodium retention. In our incident cohort this increased blood pressure has a volume component most likely due to increased dietary sodium intake as suggested by greater total peritoneal and urinary sodium losses, whereas in prevalent cohorts volume dependent hypertension may follow failure to achieve adequate peritoneal ultrafiltration and sodium removal [32]. Our study supports and association between ECW expansion and SBP.
